# *exo*,*exo*-4-(2-Hy­droxy­eth­yl)-10-oxa-4-aza­tricyclo­[5.2.1.0^2,6^]dec-8-ene-3,5-dione

**DOI:** 10.1107/S160053681200788X

**Published:** 2012-02-29

**Authors:** Xue-Jie Tan, Chen-Guang Li, Dian-Xiang Xing, Yun Liu

**Affiliations:** a School of Chemical and Pharmaceutical Engineering, Shandong Institute of Light Industry, Jinan 250353, People’s Republic of China

## Abstract

In the crystal of the title compound, C_10_H_11_NO_4_, the hy­droxy group forms an O—H⋯O_carbon­yl_ hydrogen bond with an adjacent molecule, so forming chains which extend along (010). Further weak C—H⋯O hydrogen-bonding associations give an infinite three-dimensional network structure.

## Related literature

For the first description of the title compound, see: Zhou & Chen (2000 ▶). For the synthesis of the title compound, see: Gramlich *et al.* (2010 ▶); William *et al.* (2008 ▶). For a molecular topology description, see: Braga & Grepioni (2007 ▶).
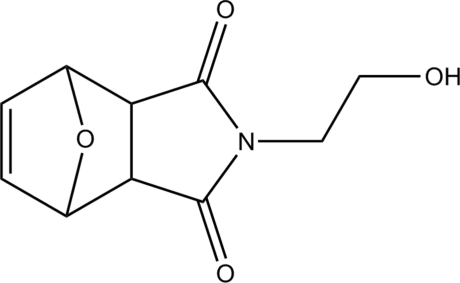


## Experimental

### 

#### Crystal data

C_10_H_11_NO_4_
*M* *_r_* = 209.20
Monoclinic, 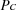

*a* = 5.4619 (12) Å
*b* = 6.8337 (15) Å
*c* = 12.546 (3) Å
β = 92.047 (3)°
*V* = 467.97 (18) Å^3^
*Z* = 2
Mo *K*α radiation
μ = 0.12 mm^−1^
*T* = 298 K
0.32 × 0.27 × 0.12 mm

#### Data collection

Bruker SMART CCD area-detector diffractometer
Absorption correction: multi-scan (*SADABS*; Bruker, 2000 ▶) *T*_min_ = 0.964, *T*_max_ = 0.986
2628 measured reflections
1017 independent reflections
999 reflections with *I* > 2σ(*I*)
*R*_int_ = 0.015

#### Refinement

*R*[*F* ^2^ > 2σ(*F* ^2^)] = 0.040
*wR*(*F* ^2^) = 0.102
*S* = 1.05
1017 reflections
141 parameters
2 restraints
H atoms treated by a mixture of independent and constrained refinement
Δρ_max_ = 0.21 e Å^−3^
Δρ_min_ = −0.25 e Å^−3^

### 

Data collection: *SMART* (Bruker, 2000 ▶); cell refinement: *SMART*; data reduction: *SAINT* (Bruker, 2000 ▶); program(s) used to solve structure: *SHELXS97* (Sheldrick, 2008 ▶); program(s) used to refine structure: *SHELXL97* (Sheldrick, 2008 ▶); molecular graphics: *SHELXTL* (Bruker, 2000 ▶) and *PLATON* (Spek, 2009 ▶); software used to prepare material for publication: *SHELXL97*.

## Supplementary Material

Crystal structure: contains datablock(s) global, I. DOI: 10.1107/S160053681200788X/zs2178sup1.cif

Structure factors: contains datablock(s) I. DOI: 10.1107/S160053681200788X/zs2178Isup2.hkl

Supplementary material file. DOI: 10.1107/S160053681200788X/zs2178Isup3.cml

Additional supplementary materials:  crystallographic information; 3D view; checkCIF report

## Figures and Tables

**Table 1:** Hydrogen-bond geometry (Å, °)

*D*—H⋯*A*	*D*—H	H⋯*A*	*D*⋯*A*	*D*—H⋯*A*
O4—H11⋯O3^i^	0.92 (6)	1.99 (6)	2.902 (3)	171 (5)
C2—H2⋯O3^ii^	0.98	2.40	3.338 (3)	160
C1—H1⋯O4^iii^	0.98	2.44	3.216 (3)	136

Symmetry codes: (i) 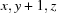; (ii) 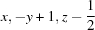; (iii) 
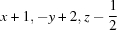
.

**Fig. 1.**
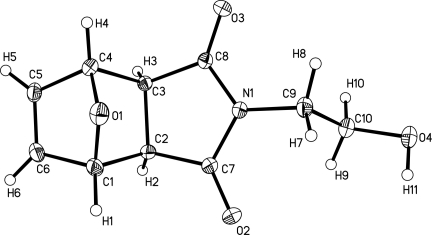
Molecular structure of (I) with atom numbering scheme with thermal ellipsoids drawn at the 30% probability level.

**Fig. 2.**
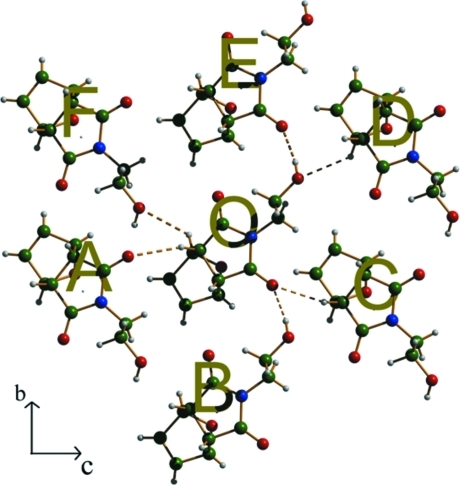
Hydrogen bonding (shown as dashed lines) in the crystal structure of (I) viewed along the a axis.

**Fig. 3.**
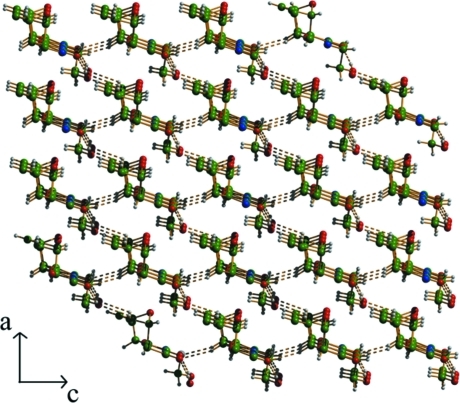
Portion of the infinite three-dimensional packing diagram of (I) viewed down the b axis.

**Fig. 4.**
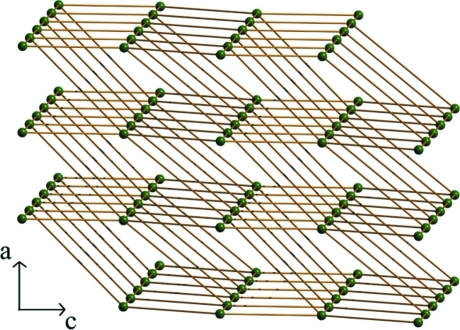
The (4.4.4.4.4.4.4.4.6.6.6.6.6.6.6.) topological diagram of (I) through abstracting every HEUNC molecule into one dummy atom viewed along the b axis.

**Fig. 5.**
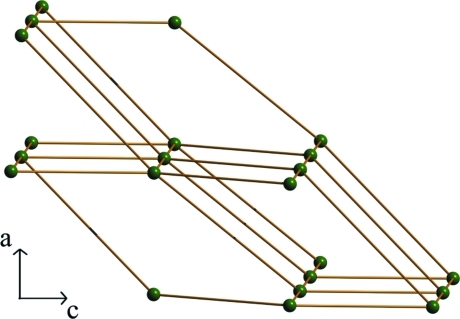
A sub-unit of the topology diagram from Fig. 4, showing ring generation from each angle intersection.

## supplementary crystallographic information

### Comment

The title compound C_10_H_11_NO_4_ (I), common name *N*-2-hydroxyethyl unsaturated norcantharidin (HEUNC), is a derivative of unsaturated norcantharidin, showing weaker anti-cancer activity than norcantharidin, possibly associated with its structure. However its single-crystal structure has not been unambiguously determined since first prepared in 2000 (Zhou & Chen, 2000). In the present work, the crystal structure of (I) is reported, and its molecular packing mode is discussed on the basis of its structure. The absolute configuration of (I) has not been determined.

The asymmetric unit of the title compound contains only one molecule (Fig. 1) in which the conformation of the *N*-substituted ethanol side chain is stabilized by intramolecular C—H···O_carbonyl_ interactions (Table 1) [torsion angles C7—N1—C9—C10 and N1—C9—C10—O4, -73.826 (12) and 168.812 (9)°], respectively. If the ethanol side chain is not considered, the molecule has a *Z*-type configuration with planes C1, C4, C5, C6 (plane 1), C1, O1, C4 (plane 2) and C2, C3, C8, N1, C7, C9, O2, O3 (plane 3) all essentially planar [dihedral angles 50.161 (8)° between planes 1 and 2 and 53.439 (7)° between planes 2 and 3].

The hydroxy group forms intermolecular O—H···O_carbonyl_ hydrogen bonds (Table 1) giving chains which extend along (010) (Fig. 2). Each molecule (shown as *O*) gives further weak C—H···O hydrogen-bonding associations with six surrounding molecules (*A*–*F*) (Fig. 2), in which molecules *B, D, E, F* and *O* are nearly co-planar, with *A* and *C* lying on either side of the plane. An overall three-dimensional network structure is formed (Fig. 3).

At first glance, the hydrogen-bonded net found in (I) may be considered distorted primitive cubic with a long vertex symbol (4.4.4.4.4.4.4.4.4.4.4.4.*.*.*.) (Braga & Grepioni, 2007) but there is a difference in connectivity along the sloping plane [Miller Index (0.74 0 0.67)] with 4-membered or 6-membered primitive rings occurring from each of the 15 (6*5/2) angles, actually giving a long vertex symbol of (4.4.4.4.4.4.4.4.6.6.6.6.6.6.6.) (Figs. 4, 5).

### Experimental

For the synthesis of the title compound, see: Gramlich *et al.* (2010); William *et al.* (2008). Elemental analysis: Calcd: C 57.41; H 5.30; N 6.70%. Found: C 57.35; H 5.23; N 6.76%.

### Refinement

The hydroxy H atom was located in a difference-Fourier map and both positional and isotropic displacement parameters were refined. Other H-atoms were included in calculated positions and were allowed to ride on the parent atom with C—H = 0.93–0.98 Å and with *U*_iso_ = 1.2*U*_eq_(C). In the absence of a suitable heavy atom in the structure, Friedel pairs (757) were merged for the final stages of refinement. The described molecule has the (C1*S*,C2*R*,C3*S*,C4*R*) relative configuration.

### Crystal data

**Table:**

C_10_H_11_NO_4_	*F*(000) = 220
*M**_r_* = 209.20	*D*_x_ = 1.485 Mg m^−^^3^
Monoclinic, *P**c*	Mo *K*α radiation, λ = 0.71073 Å
Hall symbol: P -2yc	Cell parameters from 380 reflections
*a* = 5.4619 (12) Å	θ = 2.5–28.1°
*b* = 6.8337 (15) Å	µ = 0.12 mm^−^^1^
*c* = 12.546 (3) Å	*T* = 298 K
β = 92.047 (3)°	Block, colourless
*V* = 467.97 (18) Å^3^	0.32 × 0.27 × 0.12 mm
*Z* = 2	

### Data collection

**Table:**

Bruker SMART CCD area-detector diffractometer	1017 independent reflections
Radiation source: fine-focus sealed tube	999 reflections with *I* > 2σ(*I*)
Graphite monochromator	*R*_int_ = 0.015
φ and ω scans	θ_max_ = 27.0°, θ_min_ = 3.0°
Absorption correction: multi-scan (*SADABS*; Bruker, 2000)	*h* = −6→6
*T*_min_ = 0.964, *T*_max_ = 0.986	*k* = −8→6
2628 measured reflections	*l* = −15→16

### Refinement

**Table:**

Refinement on *F*^2^	Secondary atom site location: difference Fourier map
Least-squares matrix: full	Hydrogen site location: inferred from neighbouring sites
*R*[*F*^2^ > 2σ(*F*^2^)] = 0.040	H atoms treated by a mixture of independent and constrained refinement
*wR*(*F*^2^) = 0.102	*w* = 1/[σ^2^(*F*_o_^2^) + (0.0815*P*)^2^ + 0.0262*P*] where *P* = (*F*_o_^2^ + 2*F*_c_^2^)/3
*S* = 1.05	(Δ/σ)_max_ < 0.001
1017 reflections	Δρ_max_ = 0.21 e Å^−^^3^
141 parameters	Δρ_min_ = −0.25 e Å^−^^3^
2 restraints	Extinction correction: *SHELXL97* (Sheldrick, 2008), Fc^*^=kFc[1+0.001xFc^2^λ^3^/sin(2θ)]^-1/4^
Primary atom site location: structure-invariant direct methods	Extinction coefficient: 0.049 (13)

### Special details

**Table:**

Geometry. All esds (except the esd in the dihedral angle between two l.s. planes) are estimated using the full covariance matrix. The cell esds are taken into account individually in the estimation of esds in distances, angles and torsion angles; correlations between esds in cell parameters are only used when they are defined by crystal symmetry. An approximate (isotropic) treatment of cell esds is used for estimating esds involving l.s. planes.
Refinement. Refinement of *F*^2^ against ALL reflections. The weighted *R*-factor *wR* and goodness of fit *S* are based on *F*^2^, conventional *R*-factors *R* are based on *F*, with *F* set to zero for negative *F*^2^. The threshold expression of *F*^2^ > σ(*F*^2^) is used only for calculating *R*-factors(gt) *etc*. and is not relevant to the choice of reflections for refinement. *R*-factors based on *F*^2^ are statistically about twice as large as those based on *F*, and *R*- factors based on ALL data will be even larger.

### Fractional atomic coordinates and isotropic or equivalent isotropic displacement parameters (Å^2^)

**Table:**

	*x*	*y*	*z*	*U*_iso_*/*U*_eq_	
C1	0.5015 (4)	0.6269 (3)	0.0567 (2)	0.0398 (5)	
H1	0.5792	0.7501	0.0365	0.048*	
C2	0.2181 (4)	0.6363 (3)	0.06828 (17)	0.0315 (4)	
H2	0.1262	0.6354	−0.0002	0.038*	
C3	0.1740 (3)	0.4531 (3)	0.13644 (16)	0.0278 (4)	
H3	0.0604	0.3600	0.1017	0.033*	
C4	0.4403 (4)	0.3711 (3)	0.15055 (18)	0.0343 (5)	
H4	0.4673	0.2790	0.2097	0.041*	
C5	0.5103 (4)	0.2973 (3)	0.0421 (2)	0.0398 (5)	
H5	0.5239	0.1676	0.0207	0.048*	
C6	0.5483 (5)	0.4548 (3)	−0.0159 (2)	0.0437 (6)	
H6	0.5941	0.4592	−0.0866	0.052*	
C7	0.1541 (4)	0.8058 (3)	0.13860 (18)	0.0343 (4)	
C8	0.0818 (4)	0.5321 (3)	0.24029 (17)	0.0293 (4)	
C9	0.0159 (5)	0.8636 (3)	0.3226 (2)	0.0397 (5)	
H7	0.1373	0.9666	0.3310	0.048*	
H8	0.0135	0.7907	0.3889	0.048*	
C10	−0.2353 (5)	0.9541 (3)	0.2993 (2)	0.0431 (6)	
H9	−0.2430	1.0045	0.2270	0.052*	
H10	−0.3604	0.8542	0.3048	0.052*	
N1	0.0834 (3)	0.7331 (2)	0.23622 (15)	0.0321 (4)	
O1	0.5762 (3)	0.5517 (2)	0.15900 (16)	0.0415 (4)	
O2	0.1665 (5)	0.9769 (3)	0.1182 (2)	0.0589 (6)	
O3	0.0215 (3)	0.4379 (3)	0.31657 (15)	0.0429 (4)	
O4	−0.2821 (4)	1.1065 (3)	0.37087 (18)	0.0548 (5)	
H11	−0.181 (10)	1.203 (8)	0.347 (5)	0.099 (17)*	

### Atomic displacement parameters (Å^2^)

**Table:**

	*U*^11^	*U*^22^	*U*^33^	*U*^12^	*U*^13^	*U*^23^
C1	0.0380 (12)	0.0327 (12)	0.0499 (13)	−0.0054 (8)	0.0163 (9)	−0.0014 (9)
C2	0.0376 (11)	0.0285 (10)	0.0285 (9)	0.0026 (7)	0.0043 (8)	0.0035 (8)
C3	0.0282 (9)	0.0255 (10)	0.0297 (10)	−0.0006 (7)	0.0026 (7)	−0.0006 (8)
C4	0.0352 (11)	0.0303 (10)	0.0376 (11)	0.0063 (8)	0.0026 (8)	0.0019 (8)
C5	0.0375 (11)	0.0338 (11)	0.0490 (13)	0.0053 (8)	0.0118 (9)	−0.0055 (10)
C6	0.0436 (12)	0.0421 (13)	0.0466 (13)	0.0023 (10)	0.0194 (10)	−0.0026 (11)
C7	0.0361 (9)	0.0291 (10)	0.0383 (11)	0.0017 (8)	0.0075 (8)	0.0023 (9)
C8	0.0276 (8)	0.0287 (9)	0.0315 (10)	0.0022 (7)	0.0011 (6)	−0.0008 (8)
C9	0.0433 (12)	0.0393 (12)	0.0368 (10)	0.0052 (9)	0.0034 (9)	−0.0079 (9)
C10	0.0461 (13)	0.0374 (11)	0.0463 (13)	0.0080 (10)	0.0069 (10)	−0.0047 (9)
N1	0.0342 (8)	0.0286 (8)	0.0338 (9)	0.0027 (7)	0.0056 (6)	−0.0010 (7)
O1	0.0295 (7)	0.0440 (9)	0.0509 (9)	−0.0032 (7)	−0.0010 (6)	−0.0090 (7)
O2	0.0812 (14)	0.0283 (9)	0.0692 (13)	0.0041 (9)	0.0316 (11)	0.0073 (9)
O3	0.0528 (10)	0.0418 (8)	0.0348 (8)	0.0032 (8)	0.0120 (6)	0.0078 (7)
O4	0.0668 (12)	0.0420 (10)	0.0569 (12)	0.0086 (9)	0.0218 (10)	−0.0096 (9)

### Geometric parameters (Å, º)

**Table:**

C1—O1	1.429 (3)	C5—H5	0.93
C1—C6	1.515 (3)	C6—H6	0.93
C1—C2	1.561 (3)	C7—O2	1.200 (3)
C1—H1	0.98	C7—N1	1.389 (3)
C2—C7	1.505 (3)	C8—O3	1.209 (3)
C2—C3	1.540 (3)	C8—N1	1.375 (3)
C2—H2	0.98	C9—N1	1.461 (3)
C3—C8	1.513 (3)	C9—C10	1.524 (4)
C3—C4	1.563 (3)	C9—H7	0.97
C3—H3	0.98	C9—H8	0.97
C4—O1	1.443 (3)	C10—O4	1.404 (3)
C4—C5	1.513 (3)	C10—H9	0.97
C4—H4	0.98	C10—H10	0.97
C5—C6	1.320 (4)	O4—H11	0.92 (6)
			
O1—C1—C6	102.24 (19)	C5—C6—C1	105.6 (2)
O1—C1—C2	100.56 (16)	C5—C6—H6	127.2
C6—C1—C2	106.08 (19)	C1—C6—H6	127.2
O1—C1—H1	115.4	O2—C7—N1	123.8 (2)
C6—C1—H1	115.4	O2—C7—C2	127.5 (2)
C2—C1—H1	115.4	N1—C7—C2	108.64 (18)
C7—C2—C3	104.83 (17)	O3—C8—N1	124.3 (2)
C7—C2—C1	109.78 (18)	O3—C8—C3	126.88 (18)
C3—C2—C1	101.17 (16)	N1—C8—C3	108.78 (17)
C7—C2—H2	113.4	N1—C9—C10	110.8 (2)
C3—C2—H2	113.4	N1—C9—H7	109.5
C1—C2—H2	113.4	C10—C9—H7	109.5
C8—C3—C2	104.57 (16)	N1—C9—H8	109.5
C8—C3—C4	111.56 (16)	C10—C9—H8	109.5
C2—C3—C4	101.02 (15)	H7—C9—H8	108.1
C8—C3—H3	112.9	O4—C10—C9	111.2 (2)
C2—C3—H3	112.9	O4—C10—H9	109.4
C4—C3—H3	112.9	C9—C10—H9	109.4
O1—C4—C5	101.84 (18)	O4—C10—H10	109.4
O1—C4—C3	100.13 (15)	C9—C10—H10	109.4
C5—C4—C3	106.37 (17)	H9—C10—H10	108.0
O1—C4—H4	115.5	C8—N1—C7	113.09 (18)
C5—C4—H4	115.5	C8—N1—C9	125.48 (19)
C3—C4—H4	115.5	C7—N1—C9	121.42 (18)
C6—C5—C4	105.9 (2)	C1—O1—C4	96.43 (16)
C6—C5—H5	127.0	C10—O4—H11	102 (3)
C4—C5—H5	127.0		

### Hydrogen-bond geometry (Å, º)

**Table:**

*D*—H···*A*	*D*—H	H···*A*	*D*···*A*	*D*—H···*A*
O4—H11···O3^i^	0.92 (6)	1.99 (6)	2.902 (3)	171 (5)
C2—H2···O3^ii^	0.98	2.40	3.338 (3)	160
C1—H1···O4^iii^	0.98	2.44	3.216 (3)	136
C9—H8···O3	0.97	2.58	2.911 (3)	100
C10—H9···O2	0.97	2.67	3.219 (3)	116

Symmetry codes: (i) *x*, *y*+1, *z*; (ii) *x*, −*y*+1, *z*−1/2; (iii) *x*+1, −*y*+2, *z*−1/2.
